# Phosphorylation of the Retinal Ribbon Synapse Specific t-SNARE Protein Syntaxin3B Is Regulated by Light via a Ca^2 +^-Dependent Pathway

**DOI:** 10.3389/fncel.2020.587072

**Published:** 2020-10-20

**Authors:** Joseph R. Campbell, Hongyan Li, Yanzhao Wang, Maxim Kozhemyakin, Albert J. Hunt, Xiaoqin Liu, Roger Janz, Ruth Heidelberger

**Affiliations:** ^1^Department of Neurobiology and Anatomy, University of Texas Health Science Center at Houston, Houston, TX, United States; ^2^MD Anderson Cancer Center UTHealth Graduate School of Biomedical Sciences, Houston, TX, United States

**Keywords:** syntaxin, ribbon synapse, exocytosis, bipolar cell, retina, modulation, SNARE

## Abstract

Neurotransmitter release at retinal ribbon-style synapses utilizes a specialized t-SNARE protein called syntaxin3B (STX3B). In contrast to other syntaxins, STX3 proteins can be phosphorylated *in vitro* at T14 by Ca^2+/^calmodulin-dependent protein kinase II (CaMKII). This modification has the potential to modulate SNARE complex formation required for neurotransmitter release in an activity-dependent manner. To determine the extent to which T14 phosphorylation occurs *in vivo* in the mammalian retina and characterize the pathway responsible for the *in vivo* phosphorylation of T14, we utilized quantitative immunofluorescence to measure the levels of STX3 and STX3 phosphorylated at T14 (pSTX3) in the synaptic terminals of mouse retinal photoreceptors and rod bipolar cells (RBCs). Results demonstrate that STX3B phosphorylation at T14 is light-regulated and dependent upon the elevation of intraterminal Ca^2+^. In rod photoreceptor terminals, the ratio of pSTX3 to STX3 was significantly higher in dark-adapted mice, when rods are active, than in light-exposed mice. By contrast, in RBC terminals, the ratio of pSTX3 to STX3 was higher in light-exposed mice, when these terminals are active, than in dark-adapted mice. These results were recapitulated in the isolated eyecup preparation, but only when Ca^2+^ was included in the external medium. In the absence of external Ca^2+^, pSTX3 levels remained low regardless of light/dark exposure. Using the isolated RBC preparation, we next showed that elevation of intraterminal Ca^2+^ alone was sufficient to increase STX3 phosphorylation at T14. Furthermore, both the non-specific kinase inhibitor staurosporine and the selective CaMKII inhibitor AIP inhibited the Ca^2+^-dependent increase in the pSTX3/STX3 ratio in isolated RBC terminals, while in parallel experiments, AIP suppressed RBC depolarization-evoked exocytosis, measured using membrane capacitance measurements. Our data support a novel, illumination-regulated modulation of retinal ribbon-style synapse function in which activity-dependent Ca^2+^ entry drives the phosphorylation of STX3B at T14 by CaMKII, which in turn, modulates the ability to form SNARE complexes required for exocytosis.

## Introduction

Retinal ribbon-style synapses, elaborated by the first two neurons in the visual throughput pathway, continuously convey visual information over many log units of dynamic range. These remarkable signaling abilities are supported by a number of unique features, one of which is the expression of specialized presynaptic proteins such as syntaxin3B (STX3B). STX3B is a t-SNARE protein that is expressed almost exclusively in retinal photoreceptors and bipolar cells, where it is highly enriched in the synaptic terminals (Morgans et al., 1996; Curtis et al., 2008; Liu et al., 2014). Similar to the syntaxin1 utilized by conventional synapses, STX3B interacts with SNAP-25 and VAMP/synaptobrevin to form the tripartite SNARE complex required for stimulus-evoked release of neurotransmitter release. Consistent with an essential role in exocytosis, blockade of STX3B function inhibits exocytosis from bipolar cell terminals and synaptic transmission at photoreceptor synapses (Curtis et al., 2010; Datta et al., 2017).

Syntaxin3B has a phosphorylation site at T14 in its N-terminal domain (Liu et al., 2014), a region of the molecule that may regulate SNARE complex formation and stabilization (Khvotchev et al., 2007; Rathore et al., 2010; Shen et al., 2010). This site can be *in vitro* phosphorylated by Ca^2+^/calmodulin-dependent protein kinase II (CaMKII; Risinger and Bennett, 1999; Liu et al., 2014), raising the possibility that phosphorylation at T14 *in vivo* is regulated by synaptic activity. By contrast, the comparable site in syntaxin1 of conventional synapses, S14, is not a CaMKII-substrate but is an *in vitro* substrate for casein kinase II (Risinger and Bennett, 1999) and may be constitutively phosphorylated *in vivo* (Foletti et al., 2000; Kohansal-Nodehi et al., 2016). Consistent with a functional consequence STX3B phosphorylation at T14, a STX3B T14 phosphomimetic was shown to have a higher affinity for SNAP-25 relative to wild-type STX3B (Liu et al., 2014). This has led to a model in which STX3B may not only have an essential role in neurotransmitter release at retinal-style synapses, but also a unique modulatory role governed by the activity-dependent regulation of T14 phosphorylation via CaMKII (Liu et al., 2014).

In this study, we test the hypothesis that phosphorylation of STX3B at T14 is regulated by synaptic activity in the ribbon-style synapses of the mammalian retina. Our results demonstrate for the first time that STX3 phosphorylation at T14 in the synaptic terminals of rod photoreceptors and rod bipolar cells (RBCs) is regulated by light in a Ca^2+^-dependent manner. In addition, our results confirm an *in vivo* role for CaMKII in this process and suggest that CaMKII modulates exocytosis. Together, our results establish that the retinal-specific t-SNARE protein STX3B is a phosphoprotein *in vivo* whose phosphorylation at T14 is dynamically regulated in an activity-dependent manner by CaMKII, altering neurotransmitter release.

## Materials and Methods

### Tissue Preparation

All of the animal procedures were approved by the Animal Welfare Committee of the University of Texas Health Science Center at Houston. Prior to use, adult male and female mice, strain C57BL/6J (Jackson Laboratories, Bar Harbor, ME, United States) were maintained on a standard 12 h light/dark cycle. Sacrifice was performed by cervical dislocation followed by decapitation. To minimize potential effects of circadian rhythm on syntaxin 3 (STX3) phosphorylation, animals for each set of experiments were sacrificed at the same time of day, typically between 12 and 1 pm.

#### *In vivo* and *ex vivo* Experiments

For the *in vivo* experiments, mice were unrestrained and free to move about their cage until the time of sacrifice. Two groups of mice were dark-adapted for 4 h, beginning 1 h after morning light onset. Immediately following dark-adaptation, one group of mice was sacrificed and retinal eyecups dissected free under infrared illumination and fixed, while under maintained dark conditions. The second group of mice was exposed to 15 min of a bright flashing light stimulus (3 Hz, 2,000 lux) superimposed upon a dim background light (20 lux). Animals were then sacrificed, retinal eyecups dissected free and fixed.

For the *ex vivo* experiments, two groups of mice were dark-adapted for 4 h, beginning 1 h after morning light onset. Animals were sacrificed and retinal eyecups prepared under infrared illumination. Eyecups were bathed in an oxygenated Hanks solution that contained (in mM): 128 NaCl, 5 KCl, 0.34 Na_2_HPO_4_, 0.44 KH_2_PO_4_, 10 HEPES, and 10 glucose, supplemented with either 2 CaCl_2_ and 1 MgCl_2_ (“2Ca”) or 0 CaCl_2_ and 2 MgCl (“0Ca”) pH ≈7.4, ≈315–320 mOsm. For the dark/light experiments, the left eyecup from each animal was maintained under dark-adapted conditions and then fixed. The right eyecup was exposed to 15 min of a bright flashing light stimulus (3 Hz; 1000 lux) superimposed on a dim light background (20 lux) and then fixed. Similarly, for the 2Ca/0Ca experiments, one eyecup from each animal was placed in the 2Ca external solution and the other in the 0Ca solution.

#### Isolated Rod Bipolar Cells

Rod bipolar cells were dissociated from the retinae of light-adapted mice by enzymatic digestion followed by mechanical trituration (Heidelberger and Matthews, 1992; Zhou et al., 2006) and plated onto poly-L-lysine-coated coverslips. Cells were allowed to settle and attach for 20–30 min. During this time, they were bathed in oxygenated solution containing either (in mM): 152 NaCl, 2.6 KCl, 1.3 MgCl_2_, 0.5 CaCl_2_, and 10 HEPES, or 153 NaCl, 2.6 KCl, 3.0 MgCl_2_, 0 CaCl_2_, and 10 HEPES (“low Ca”). Following the settling phase, coverslips were either incubated for an additional 30 min in the low Ca solution or incubated in a “high Ca” solution that was designed to elevate the intraterminal free Ca^2+^ concentration. The latter had a reduced Na^+^ concentration, a perturbation that we have previously shown activates the reverse mode of the Na^+^/Ca^2+^ exchanger in the RBC synaptic terminal to produce a modest, sustained elevation in the spatially-averaged intraterminal free Ca^2+^ concentration (Wan et al., 2012). The “high Ca^2+^” solution contained (in mM): 30 NaCl, 123 choline chloride, 2.6 KCl, 1 MgCl_2_, 2.0 CaCl_2_, and 10 HEPES. Kinase inhibitors were applied during the settling and incubation phases. For all, the solution pH was adjusted to ≈7.4 and the osmolarity was 290–300 mOsm. Following treatment, cells were fixed for 10 min in 4% formaldehyde.

### Tissue Processing and Immunolabeling

#### Retinal Sections

Retinal eyecups were fixed for 10 min at room temperature in freshly prepared 4% formaldehyde, rinsed with PBS, cryoprotected in 30% sucrose and stored at 4°C overnight. Processing of retinal eyecups was similar to that described previously (Kothmann et al., 2007; Li et al., 2009, 2013). In brief, cryoprotected eyecups were embedded in Tissue-Tek O.C.T. compound (Sakura Finetek, Torrance, CA, United States), fast frozen at −20°C and sliced into 30 μm cryostat sections. Sections were blocked for 1 h in PBS supplemented with 5% donkey serum and 0.3% Triton and then incubated overnight at room temperature in PBS supplemented with 1% donkey serum, 0.3% Triton and primary antibodies (see below). Sections were then washed thoroughly with PBS and incubated in PBS containing the secondary antibodies (see below) for 2 h at room temperature. Sections were then washed and mounted with Prolong Gold anti-fade mounting medium (Thermo Fisher Scientific, Waltham, MA, United States). Slides were stored in a light protective holder at 4°C before imaging.

#### Isolated Rod Bipolar Cells

Fixed cells were blocked for 2 h at room temperature in PBS supplemented with 5% donkey serum and 0.4% Triton X-100 and then incubated overnight at 4° in PBS supplemented with 2% donkey serum, 0.4% Triton X-100 and primary antibodies (see below). After washing in PBS, cells were then incubated for 2 h in PBS containing the secondary antibodies (see below). Cover slips were washed and mounted with Prolong Gold anti-fade with DAPI mounting medium (Thermo Fisher Scientific, Waltham, MA, United States). Slides were stored in a light protective holder at 4°C before imaging.

#### Antibodies

Syntaxin 3 was immunolabeled with a mouse-monoclonal antibody initially received as a generous gift from Dr. Muna Naash (Zulliger et al., 2015) and then purchased from EMD Millipore (Burlington, MA, United States), clone 12E5. This antibody was generated against the cytoplasmic portion of mouse syntaxin 3B (STX3B; amino acids 2–270). However, the two cytoplasmic parts of the retinal specific isoform STX3B and the non-neuronal splice form syntaxin 3A (STX3A) differ only in one exon and are 93% identical. We independently verified that this antibody is selective for STX3 and recognizes both STX3A and STX3B isoforms (Supplementary Figure 1). Given that STX3A is not expressed at detectable levels in the mouse retina (Curtis et al., 2008) the observed immunolabeling of synapses most likely corresponds to STX3B. We further validated the 12E5 antibody by demonstrating that a phospho-mimetic mutation (T14E) of STX3B does not prevent binding of the 12E5 antibody and that a STX3 antibody raised against an epitope located in the N-terminus (amino acids 2–17) does not occlude 12E5 binding. These findings indicate that the unidentified epitope recognized by the 12E5 STX3 antibody is not located within the N-terminal region of STX3, where T14 resides. To perform quantitative immunolabeling, saturation of binding sites is desirable, and therefore the STX3 antibody was used at a dilution of 1:200 in tissue sections and at 1:400 in dissociated retina. Phosphorylated-syntaxin3 at T14 (pSTX3) was immunolabeled with an affinity purified rabbit polyclonal antibody that we have generated and extensively characterized previously [UT 649, (Liu et al., 2014)] used at a dilution of 1:200 in retinal sections and 1:300 for isolated cells. Cones were identified by immunolabeling with an arrestin antibody Arrestin-C (1–17), goat polyglonal IgG (Santa Cruz Biotechnology, Dallas, TX, United States) at 1:200 dilution. RBCs were identified by immunolabeling with a PKCα antibody (C-20; sc-208-G, goat-polyclonal IgG; Santa Cruz Biotechnology, Dallas, TX, United States) at a concentration of 1:400 in tissue sections or 1:1000 in dissociated retina. In experiments in which pCaMKII was immunolabeled, a rabbit-polyclonal IgG pCaMKII T286/287 antibody, which recognizes all four isoforms of CaMKII phosphorylated at T286/287 (Invitrogen, Carlsbad, CA, United States) was used at 1:250. All secondary antibodies were obtained from Jackson Immuno-Research Labs (West Grove, PA, United States). Cy3-conjugated anti-mouse IgG and Alexa 488-conjugated anti-rabbit IgG were used at a dilution of 1:200 in tissue sections, and anti-goat Alexa 488, anti-rabbit Alexa 594, and anti-mouse Alexa 647 were used at a dilution of 1:400 in dissociated retina. In some experiments on dissociated retina, anti-rabbit Alexa 594 was replaced with Anti Rabbit Cy3 at 1:400 with no differences in outcome noted.

### Quantitative Confocal Microscopy

#### Retinal Sections

Image acquisition and data analysis were conducted in similar manner to that described previously (Kothmann et al., 2007; Li et al., 2009). Rod spherules, cone pedicles and RBC terminals in retinal sections were identified by their characteristic appearance and respective locations within the outer plexiform layer (OPL) or inner plexiform layer (IPL) and by immunolabeling for STX3 (Sherry et al., 2006; Liu et al., 2014). Images of phospho-syntaxin3 at T14 and syntaxin3 signals were acquired on a Zeiss 780 confocal microscope using standardized settings to facilitate the comparison of labeling across experiments and animals. The brightest pixel intensity for each channel was slightly saturated so that the intensity of the majority of pixels fell in the linear range of the intensity curve. For the *in vivo* and *ex vivo* experiments, quantification of the pSTX3 and STX3 signals was obtained from the analysis of five images from two different histological sections from each eyecup at 0.5 μm intervals from randomly chosen locations 50 to 80% from the distance of the optic nerve head to the periphery.

Tissue sections were analyzed using the ROI function of SimplePCI software (versions 5.3 and 6.0, Hamamatsu; Hamamatsu Photonics, Bridgewater, NJ, United States) that allowed for the manual selection of ROIs based on STX3 immunolabeling. ROIs were defined as having a STX3 immunoreactivity that was greater than 20% threshold of the total intensity range. ROIs representative of rod spherules were selected based upon STX3 immunolabeling, location in the upper OPL, round shape and smaller size relative to cone pedicles, and the lack of arrestin-labeling. ROIs representative of cone pedicles were chosen based on the large, STX3-positive plaques associated with cone-arrestin and a location at the bottom of OPL. ROI’s representative of RBC terminals were identified based upon STX3 immunoreactivity, size and location at the bottom of the IPL. For each OPL image, data from ≈30–60 rod terminals and ≈5–15 cone terminals were obtained. For each IPL image, data from ≈25 RBC terminals were obtained. The mean pixel intensity values from each ROI in an image were collected and the pSTX3/STX3 ratio per ROI calculated. The individual ratios from a given image were not normally-distributed, and therefore the median value rather than the mean was used to denote the pSTX3/STX3 ratio representative of each image. The value per animal or eyecup was calculated as the mean of the representative image values, and the value across animals or eyecups per condition was calculated as the average of these values and expressed as the mean ± s.e.m. As such, the value “n” denotes the number of animals or eyecups per condition rather than the number of terminals. Results were compared across the two conditions (e.g., light/dark, 0/2 mM Ca^2+^) using independent sample *t*-tests. Tissue from multiple (2–4) animals was sampled per experimental condition. Figure images have been cropped and are displayed as maximal intensity projections.

#### Isolated Rod Bipolar Cells

Methods for adjustment of confocal settings and image acquisition were similar to those described above for retinal sections. Isolated RBC were identified by their characteristic morphology and PKC immunoreactivity (Negishi et al., 1988; Wan et al., 2008). A minimum of 10 (range ≈ 10–30) isolated RBC terminals were analyzed per experimental condition per mouse. Quantification of the ratio of phospho-syntaxin3 at T14 to syntaxin3 (pSTX3/STX3) was performed in ImageJ 1.50i (NIH) using two different approaches. The first used thresholding of the syntaxin 3 signal in the RBC terminal to identify the ROI for the comparable phospho-syntaxin 3 measurement. For the second, two data analyzers, blinded to experimental conditions, collected data from ROIs hand-drawn around PKCα positive synaptic terminals. The two sets of analyses gave similar results, and the results of the latter are presented. For each experimental condition, the mean of the phospho-syntaxin3 to syntaxin3 ratio per mouse is presented. As a ratiometric approach could not be applied to quantification of pCaMKII immunoreactivity, results were reported as mean pixel intensities or as percent change from baseline. The latter allowed for better comparison across experimental replications and was calculated as [(V2−V1)/V1)] × 100, where V_1_ was the mean pixel intensity in the low Ca^2+^ condition and V_2_ was the mean pixel intensity of the high Ca^2+^ condition with or without inhibitor. The percent change was then averaged across animals to determine the percent change from baseline per group. Statistical analyses were performed using Prism 7 (Graph Pad, San Diego, CA, United States). Normality of the data was verified using column statistics. Two-tailed independent sample *t*-tests and One-Way ANOVA followed by Tukey’s posthoc multiple comparisons test were used as appropriate. Data are expressed as mean ± s.e.m, where *n* = the number of mice per condition. For immunocytochemistry on isolated RBCs, tissue from multiple (3–7) animals was sampled per experimental condition. Figure images are displayed as maximal intensity projections.

### Electrophysiology

To measure exocytosis from RBCs, retinal slices (≈200 μm thick) were prepared under dim red light illumination using a handmade, semiautomatic chopper. The external bath solution (bubbled with a mixture of 95% O_2_ and 5%CO_2_) contained (in mM): 125 NaCl; 1.2 CaCl_2_; 0.5 MgCl_2_; 1.5 KCl; 1.3 NaH_2_PO_4_; 10 D-Glucose; 23 NaHCO_3,_ with pH 7.3, 294–296 mOsm. Retinal slices were allowed to rest in the dark for ≥1 h under continuous perfusion prior to use. Slices were transferred under dim light to the recording chamber. The external bath solution used for recordings was identical to that above except that it additionally contained (in μM): 100 picrotoxin, 50 μM 1,2,5,6-Tetrahydropyridin-4-yl methylphosphinic acid (TPMPA), 10 strychnine to block GABA_A_, GABA_C_, and glycine receptors, respectively. To minimize artifacts in the capacitance record associated with perfusion, bath perfusion was halted during capacitance measurements.

Membrane capacitance was measured in whole-cell recording mode with an 8–12 MΩ borosilicate pipette placed on the soma of an intact RBC. The control internal pipette solution contained (in mM): 100 Cs-Methanesulfonate; 20 TEA-Cl; 0.2 MgCl_2_; 10 phosphocreatine-Na_2_; 0.5 Na-GTP; 1 mM EGTA, 20 HEPES, pH = 7.2, 286–290 mOsm. To test the effects of CaMKII inhibition on RBC exocytosis, the selective CaMKII inhibitor AIP was added to the internal recording solution to achieve a final concentration of 25 μM (Sakaba and Neher, 2001; Tatone et al., 2002; Gao et al., 2006; Mockett et al., 2011). Measurement of baseline parameters began 10–15 min after achieving the whole-cell recording configuration to allow time for reagents to reach stable levels within the synaptic terminal. To confirm the entry of Cs^+^ and TEA^+^ into the synaptic compartment, a linear voltage ramp (−65 mV to +20 mV, 24.2 mV/s) was applied approximately 1 min prior to the depolarizing pulse train stimulus and the current–voltage relationship evaluated for efficacy of K^+^ channel blockade and isolation of the presynaptic, L-type Ca^2+^ current (Heidelberger and Matthews, 1992; Heidelberger et al., 2005; Wan and Heidelberger, 2011). Exocytosis was driven by a train of ten depolarizing voltage steps (−65 to −20 mV) given at 4 Hz (Wan et al., 2010).

Membrane capacitance measurements were made with the use of an EPC-10 patch-clamp amplifier and Patchmaster software (v3.92 Heka Electronics, Lambrecht, Germany). In brief, a 500-Hz, 20-mV peak-to-peak sinusoidal voltage stimulus was superimposed on a holding potential of −65 mV (Zhou et al., 2006). The resultant signal was processed using the Lindau-Neher technique to yield estimates of membrane capacitance, C_m_, and membrane conductances G_m_ and G_s_. For each 100 ms sine wave segment, the mean values for C_m_, G_m_, and G_s_ were recorded. Measurement of C_m_ was temporally halted during a depolarizing voltage step and for the first 25 ms thereafter to minimize the confounding contributions of current flow and channel gating to C_m_ (Horrigan and Bookman, 1994; Wan et al., 2008, 2010). RBCs were included in the final data set if they had an access resistance of less than ≈75 MΩ and a well-isolated, measurable Ca^2+^ current. Control and AIP-exposed cells in the final data set were similar with respect to the mean resting access conductance, G_s_, (control: 2.06e-8 ± 2.04e-9S; AIP: 1.86e-8 ± 1.71e-9S; *p* = 0.466, *n* = 6,5) and the mean resting membrane conductance, G_m_, (control: 1.46e-10 ± 5.2e-11S; AIP: 5.0e-11 ± 1.6e-11S; *p* = 0.138; *n* = 6,5 cells). In addition, there was no statistical difference in the resting C_m_ (control: 6.48 ± 0.811 pF; AIP: 4.61 ± 0.385 pF; *p* = 0.083, *n* = 6,5) or in the standard deviation of the resting C_m_ signal (baseline noise) (control 11.4 ± 2.37 fF; AIP: 9.31 ± 1.74 fF; *p* = 0.494. *n* = 6,5). Data were exported to IGOR Pro (v6.3.7.2, WaveMetrics) for analysis of membrane current and C_m_, G_m_, and G_s_. Two-tailed independent sample *t*-tests with or without Welch’s correction were used, as appropriate. Statistical analyses were performed in Excel (Microsoft, Redmond, WA, United States) and Prizm 7 (GraphPad Software, Inc., San Diego, CA, United States).

### Chemicals

Staurosporine (ThermoFisher, Waltham, MA, United States) was made as a 2.15 mM stock solution in DMSO and used at a final concentration of 100 nM. KN93 (Selleckchem, Houston, TX, United States) was made up as a 3.35 mM stock solution in MQ water and used at a final concentration of 1 μM. KN92 (Biovision, Milpitas, CA, United States) was made up as a 3.6 mM stock in DMSO and used at a final concentration of 1 μM. Myristoylated-AIP (mAIP; Tocris, Bristal, United Kingdom), a membrane permeant analog of the CaMKII inhibitor AIP (Autocamtide-2-related inhibitory peptide), was made as a 293 μM stock solution in MQ water and used at a final bath concentration of 100 nM. In the isolated cell experiments, drugs were added to the external bath solution. For drugs made up in DMSO, addition of DMSO to the bath solution at the same concentration as in the test condition had no effect on pSTX3B or pCaMKII levels (not shown). For electrophysiological measurement of exocytosis, a 1 mM stock solution of AIP (Tocris, Bristal, United Kingdom) was made in MQ water and added to the internal pipette solution at a final concentration of 25 μM (Sakaba and Neher, 2001; Tatone et al., 2002; Gao et al., 2006; Mockett et al., 2011). All other chemicals were purchased from Sigma.

## Results

### Phosphorylation of STX3B at T14 Is Regulated by Light

We have shown previously that in the mammalian retina, STX3 can be phosphorylated at T14 under physiological conditions (Liu et al., 2014). Furthermore, *in vitro* biochemical studies have shown that T14 of STX3 is phosphorylated by Ca^2+^/CaMKII (Risinger and Bennett, 1999; Liu et al., 2014). This raises the possibility that phosphorylation at this site *in vivo* may be regulated by synaptic activity. In the first part of the present study, we ask whether phosphorylation of STX3 at T14 in the mammalian retina is regulated *in vivo* by light.

Levels of STX3 phosphorylated at T14 (pSTX3) and total STX3 levels in the synaptic terminals of rod and cone photoreceptors and RBCs were measured using quantitative immunofluorescence. To this end, retinal sections were double-labeled with a phospho-specific antibody that recognizes pSTX3, which we previously developed and extensively characterized (Liu et al., 2014) and a STX3 antibody (Zulliger et al., 2015) that we further characterized as part of this study. As shown in Supplementary Figure 1, the latter recognizes both STX3 and a STX3 T14 phosphomimetic mutant and is not occluded by the binding of an antibody raised against an epitope in the N-terminus of STX3 that encompasses T14. This pair of antibodies was used to provide a read-out of pSTX3 and STX3, respectively, which in retinal ribbon-style synapses, is indicative of pSTX3B and STX3B (Curtis et al., 2008). The retinae from dark-adapted mice and dark-adapted mice that were subsequently exposed to 15 min of a light stimulus were compared. Data were quantified by determining the ratio of pSTX3 to total STX3 of individual synaptic terminals of rod and cone photoreceptors and rod bipolar cells for each condition. For each animal, results were compiled across several retinal sections for each type of synaptic terminal and a single composite data point per type of synaptic ending point per animal reported (see section “Materials and Methods”).

**FIGURE 1:**
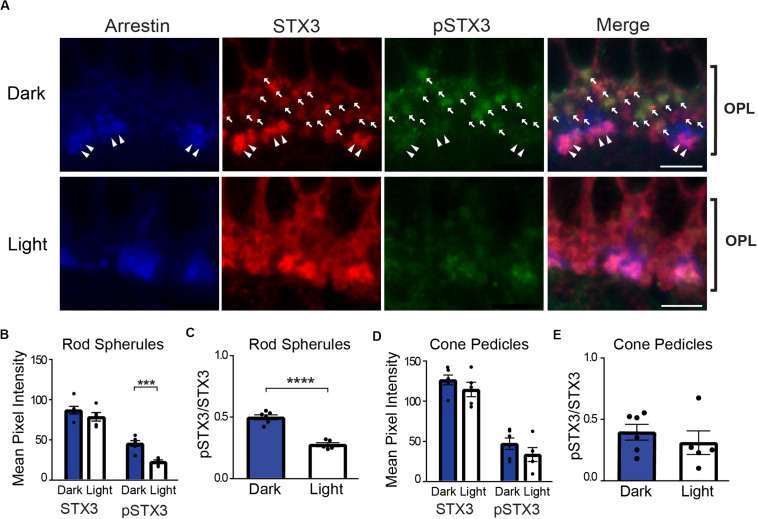
In rod terminals, phosphorylation of STX3 at T14 is greater in dark-adapted mice than in light-exposed mice. **(A)** Top: A representative set of confocal images of a vertical section through the outer plexiform layer (OPL) of the retina of a dark-adapted mouse. Arrows mark the presumptive rod terminals in the image. Note that many of STX3-labeled rod terminals are also pSTX3 positive. Double arrowheads mark the arrestin-positive cone terminals. Bottom: A representative set of confocal images through the OPL of retina from a dark-adapted mouse exposed to a 15-min light stimulus. Note the relatively weak pSTX3 immunoreactivity. For upper and lower panels, retinal sections were triple-labeled for cone-arrestin (blue) to mark the cone terminals, STX3 (red), which labels both rod and cone terminals, and pSTX3 (green). Scale bar = 5 μm. **(B)** Comparison of the mean pixel intensity of STX3 immunolabeling in rod terminals revealed no light/dark difference. By contrast, the mean pixel intensity of pSTX3 in rod terminals was significantly diminished in the light when compared to the dark (*p* = 0.0006). **(C)** Quantification of the pSTX3 to STX3 ratio in rod terminals demonstrates that in the dark-adapted mouse (blue bars) the mean ratio of pSTX3 to STX3 is significantly greater than in the light-treated mouse (white bars). *p* < 0.0001. **(D)** There was no light/dark difference in the mean pixel intensity of STX3 immunolabeling in cone terminals and no clear light/dark difference in pSTX3 immunolabeling. **(E)** Quantification of the pSTX3 to STX3 ratio in cone terminals indicated a mean pSTX3 to STX3 ratio that is not significantly different between lighting conditions. *p* = 0.465. For **(B–E)**, *n* = 6,5 (mice). ***Indicates *p* < 0.001 and ****indicates *p* < 0.0001.

Figure 1A depicts a set of representative confocal images of the OPL of vertical retinal sections from a dark-adapted mouse (top) and from a dark-adapted mouse exposed to 15 min of a light stimulus (bottom) that are triple-labeled for cone arrestin (blue), STX3 (red), and pSTX3 (green). A strong pSTX3 signal is evident in the section from the dark-adapted mouse (Figure 1A, top) relative to the light-exposed animal (Figure 1A, bottom). Quantitation of the results in rod terminals from multiple histological sections and animals demonstrated that total STX3 levels, expressed as mean pixel intensity, were virtually identical in the dark-adapted and light-exposed mice (Figure 1B: dark: 87 ± 5 vs. light: 79 ± 5; *p* = 0.2673, *t* = 1.182, and *n* = 6,5). By contrast, pSTX3 levels were approximately two times higher in the dark-adapted mice than in light-exposed mice (Figure 1B; dark: 46 ± 4 vs. light: 23 ± 2; *p* = 0.0006, *t* = 5.207). These data suggest a selective, light-driven decrease in the phosphorylation of STX3 at T14. To quantify the change in STX3 phosphorylation, we calculated the pSTX3/STX3 ratio for each condition. Results show that the pSTX3 to STX3 ratio in rod terminals was nearly two times higher in the dark-adapted mouse, when rods are depolarized and releasing neurotransmitter, as compared to the light-exposed mouse, when rods are expected to be quiescent (Figure 1C; dark: 0.497 ± 0.020 vs. light: 0.276 ± 0.016; *p* < 0.0001, *t* = 8.372, and *n* = 6,5). Cone terminals, on the other hand, showed little evidence of a light-regulated change in the pSTX3 to STX3 ratio (Figures 1D,E). This may reflect the broader operational range of cone photoreceptors and methodological limitations (see section “Discussion”).

**FIGURE 2:**
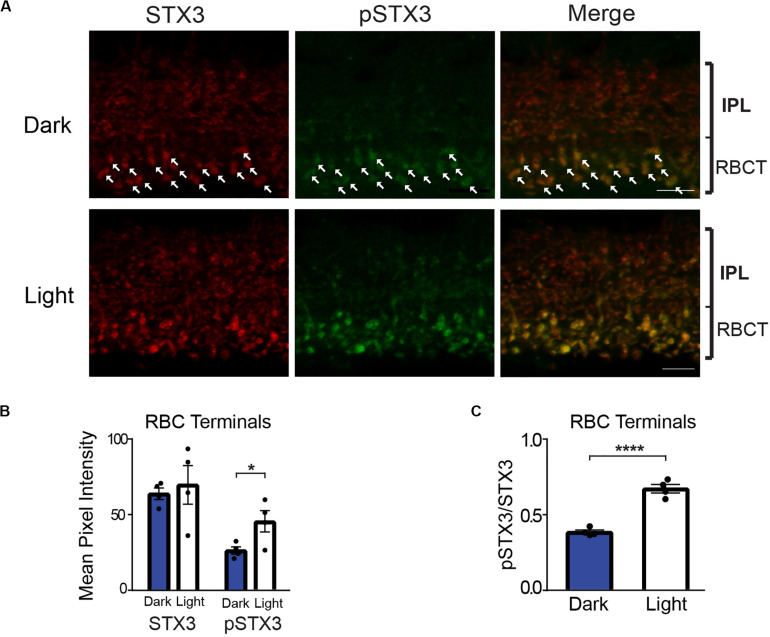
In rod bipolar cell terminals, phosphorylation of STX3 at T14 is greater in light-exposed mice than dark-adapted mice. **(A)** Top: A representative set of confocal images of a vertical section through the inner plexiform layer (IPL) of a dark-adapted mouse retina. Arrows mark some of the rod bipolar cell synaptic terminals. Bottom: A representative set of confocal images through the IPL of a retina from a dark-adapted mouse exposed to a 15-min light stimulus. Note the obvious double-labeling for pSTX3 and pSTX3 in rod bipolar cell terminals. For all, retinal sections were doubled-labeled for STX3 (red), and pSTX3 (green). The small bracket labeled “RBCT” indicates the region of the IPL where the rod bipolar cell terminals (RBCT) reside. Scale bar = 10 μm. **(B)** Quantification of the rod bipolar cell terminal mean pixel intensities demonstrates that only the mean intensity of pSTX3 immunolabeling is light-sensitive *p* = (0.0415). Quantification of the pSTX3 to STX3 ratio in rod bipolar cells terminals demonstrates that in the light-treated mouse (white bars) the ratio of pSTX3 to STX3 is significantly greater in the dark-adapted mouse (blue bars). *p* < 0.0001. For **(B,C)**, *n* = 4,4 (mice). *Indicates *p* < 0.05 and ****indicates *p* < 0.0001.

Figure 2 shows a representative set of confocal images of vertical retinal sections taken of the IPL double-labeled for STX3 (red) and pSTX3 (green). In the dark-adapted state, a modest level of pSTX3B labeling can be observed at the bottom of the proximal inner plexiform layer, where the synaptic terminals of RBCs reside (Figure 2A, top). The level of pSTX3 labeling in this region is enhanced following light exposure (Figure 2A, bottom). Quantification of multiple experiments revealed that pSTX3B to STX3B ratio is significantly increased in RBC synaptic terminals of light-exposed mice relative to those of dark-adapted mice (Figure 2C; light: 0.672 ± 0.028 vs. dark: 0.385 ± 0.013; *p* < 0.0001, *t* = 9.258; *n* = 4,4 mice). Furthermore, the light-dependent increase in pSTX3 labeling does not arise from a light-regulated change in total synaptic STX3 (Figure 2B; dark: 64 ± 4 vs. light: 69 ± 13; *p* = 0.6747, *t* = 0.4409) but rather a specific light-dependent increase in pSTX3B (Figure 2B: dark: 26 ± 2 vs. light: 46 ± 7; *p* = 0.0415, *t* = 2.584) Given that RBCs are depolarized by light, these findings suggest that pSTX3 levels are higher in depolarized terminals relative to quiescent ones. Taken together, results obtained rod photoreceptors and rod bipolar cells indicate a correlation between periods of neuronal activity and the phosphorylation of STX3 at T14.

### Light-Regulated Phosphorylation of STX3B at T14 Requires External Ca^2+^

We next asked whether external Ca^2+^, which enters nerve terminals during synaptic activity, was required for phosphorylation of STX3 at T14. To this end, eyecups were isolated from dark-adapted mice and bathed in our standard, Ca^2+^-containing bath solution or in a nominally Ca^2+^-free bath solution. Isolated eyecups were then subjected to either an additional 15 min of darkness or a light stimulus for 15 min.

**FIGURE 3:**
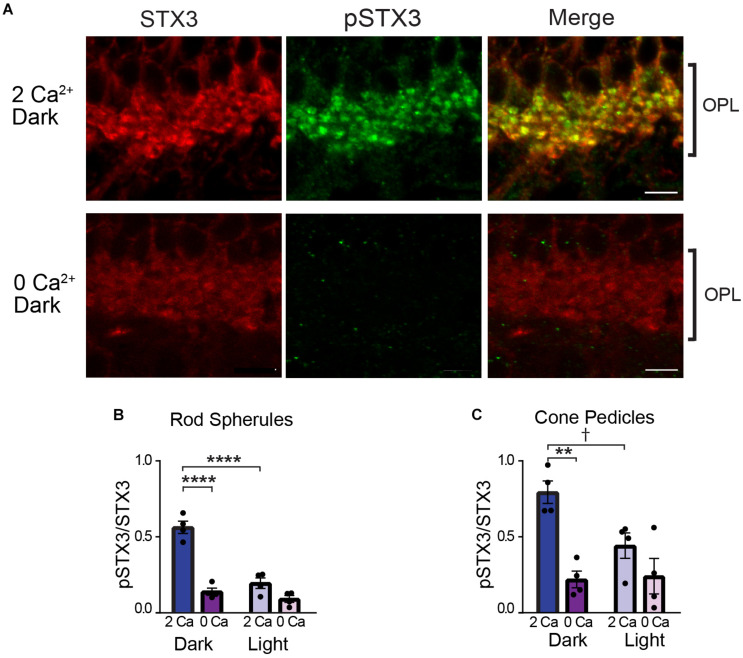
External Ca^2+^ is required for STX3 phosphorylation at T14 in both rod and cone photoreceptor terminals. **(A)** Representative confocal images through the OPL show that phosphorylation of STX3 at T14 in photoreceptors terminals in dark-adapted retinal eyecups (top panel) is abolished by the omission of Ca^2+^ from the external bath solution (“O Ca,” bottom panel). Scale bar = 5 μm. **(B)** In rod spherules, the pSTX3/STX3 ratio in dark-adapted retinal eyecups was greatly reduced in the absence of external Ca^2+^ (*p* < 0.0001) and indistinguishable from that of light-treated eyecups, with or without external Ca^2+^; *p* = 0.6035 and 0.6928, respectively. **(C)** In cone pedicles, the high pSTX3/STX3 ratio in dark-adapted eyecups was reduced in the absence of external Ca^2+^ to levels indistinguishable from that of light-treated eyecups, with or without external Ca^2+^; *p* = 0.3016; *p* = 0.998, respectively. For **(B,C)**, *n* = 4,4 (mice). **indicates *p* < 0.01, ****Indicates *p* < 0.0001, and ^†^indicates that *p* approaches significance.

As shown in Figure 3, in rod terminals under normal Ca^2+^ conditions, we saw a significantly higher pSTX3 to STX3 ratio in the dark than in the light (Figure 3B; dark: 0.563 ± 0.040 vs. light: 0.196 ± 0.035; *n* = 4,4; *p* < 0.0001), recapitulating the results observed in the live mouse experiments. This dark-driven phosphorylation of STX3B at T14 in rod terminals (Figure 3A, top) was prevented when the eyecup was bathed Ca^2+^-free external solution (Figure 3A, bottom). Quantification of multiple experiments revealed that the pSTX3/STX3 ratio of rod terminals in dark-adapted eyecups was approximately 2.5 times larger in the presence of external Ca^2+^ than in its absence (Figure 3B; 0.563 ± 0.040 vs. 0.140 ± 0.023; *n* = 4,4; *p* < 0.0001). In the light-exposed eyecup, removal of external Ca^2+^ did not further reduce the pSTX3 to STX3 ratio (Figure 3B 0.140 ± 0.023 vs. 0.092 ± 0.023, *n* = 4,4, *p* = 0.1374).

In cone terminals, the ability of the light stimulus to reduce the pSTX3 to STX3 ratio attained in the dark-adapted state under normal Ca^2+^ conditions approached significance (Figure 3C; dark: 0.796 ± 0.074 vs. light: 0.445 ± 0.084; *n* = 4,4; *p* = 0.0551), possibly reflecting the greater ease with which isolated eyecups could be oriented to face the light stimulus relative to live mice. Importantly, removal of external Ca^2+^ significantly reduced the dark-adapted levels of STX3 phosphorylation at T14 (Figure 3C; 0.796 ± 0.074 vs. 0.222 ± 0.055; *n* = 4,4; *p* = 0.0023), indicating that cone terminals also exhibit Ca^2+^-dependent STX3 phosphorylation at T14. There was no further decrease in the pSTX3 to STX3 ratio in photoreceptor terminals in light-exposed eyecups in the absence of external Ca^2+^ (Figures 3B,C; rods: 0.140 ± 0.023 vs. 0.092 ± 0.023, *n* = 4,4, *p* = 0.1374; cones: 0.222 ± 0.055 vs. 0.243 ± 0.116, *n* = 4,4 *p* = 0.3802).

**FIGURE 4:**
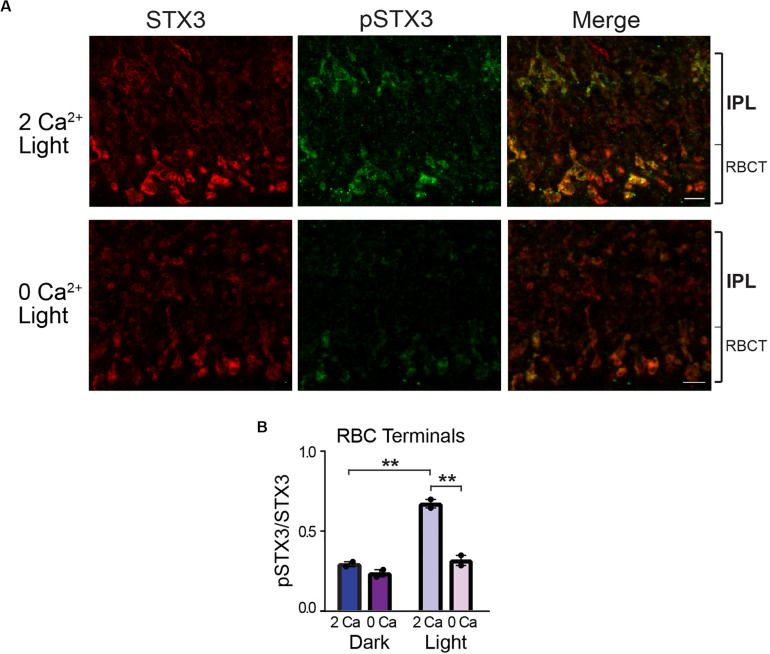
External Ca^2+^ is required for STX3 phosphorylation at T14 in rod bipolar cell synaptic terminals. **(A,B)** A pair of representative confocal images show that phosphorylation of STX3 at T14 in rod bipolar cell synaptic terminals in light-treated retinal eyecups is reduced by the omission of Ca^2+^ from the external bath solution (“O Ca”). The small bracket labeled “RBCT” indicates the region of the IPL where the rod bipolar cell terminals reside. Scale bar = 5 μm. **(B)** In rod terminals, the pSTX3/STX3 ratio in dark-adapted retinal eyecups was greatly reduced in the absence of external Ca^2+^ (*p* = 0.0019) and indistinguishable from that of dark-adapted eyecups, with or without external Ca^2+^; *p* = 0.9043 and 0.2382, respectively. *n* = 2,2 (mice). **Indicates *p* < 0.01.

Rod bipolar cells are depolarized by light. Therefore, if phosphorylation of STX3B at T14 requires activity-dependent Ca^2+^ entry, then in RBC terminals, phosphorylation levels should be highest in light-exposed eyecups when external Ca^2+^ is present and remain at or near baseline levels when in the dark or when bathed in a Ca^2+^-free solution in the light. The results depicted in Figure 4 supports this prediction. In the presence of external Ca^2+^, RBC terminals in light-stimulated eyecups exhibited an approximately two-fold higher pSTX3B to STX3B ratio relative to those of dark-adapted eyecups (Figure 4B; 0.673 ± 0.027 vs. 0.295 ± 0.150, *p* = 0.0014, *n* = 2,2). That external Ca^2+^ was required for this light-driven doubling of the pSTX3 to STX3 ratio was evidenced by the virtual abolition of the light-driven increase in Ca^2+^-free external solution (0.673 ± 0.027 vs. 0.318 ± 0.032, *p* < 0.0019, *n* = 2,2) and maintenance of the pSTX3 to STX3 ratio at a level indistinguishable from that observed in the dark-adapted eyecup in the absence of Ca^2+^ (0.318 ± 0.032 vs. 0.238 ± 0.223, *n* = 2,2 *p* = 0.238). Taken together, the results of this set of experiments demonstrate that the light-regulated phosphorylation of STX3 on T14 in synaptic terminals of retinal photoreceptor and rod bipolar cells is dependent upon external Ca^2+^, which presumably enters the synaptic terminals via voltage-gated Ca^2+^ channels during synaptic activity (Heidelberger et al., 2005; Wan and Heidelberger, 2011).

### Elevation of Intraterminal Ca^2+^ Drives STX3B Phosphorylation

**FIGURE 5:**
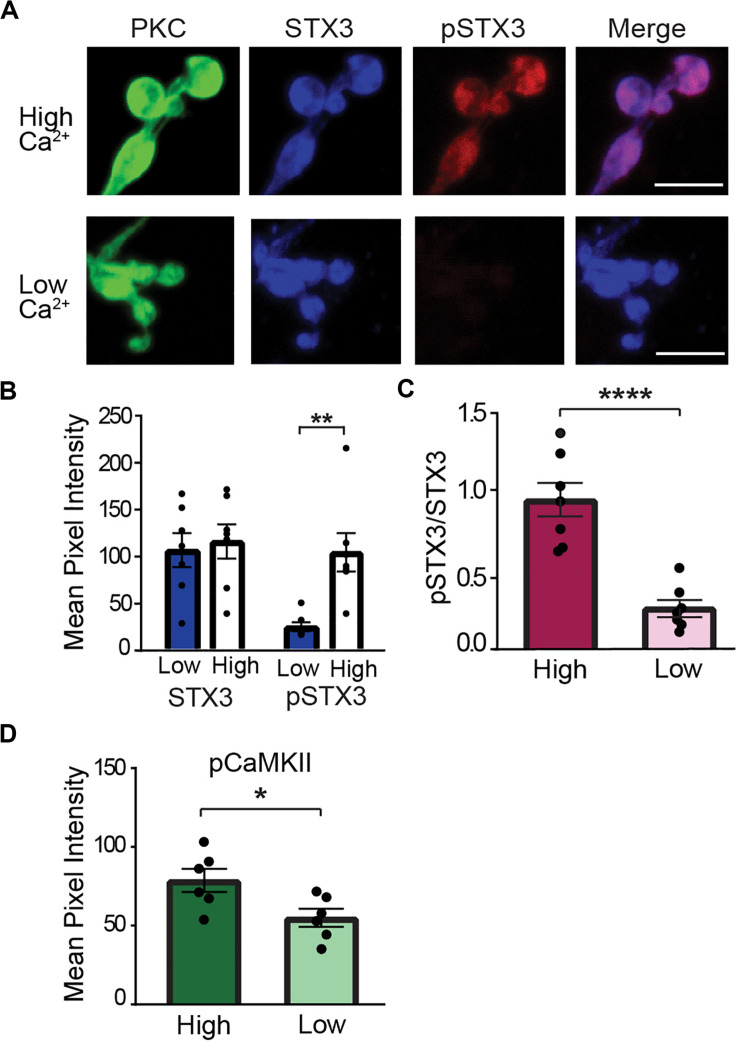
Elevation of intraterminal Ca^2+^ promotes phosphorylation of STX3B at T14 in rod bipolar cell synaptic terminals. **(A)** Confocal images of synaptic terminals of isolated rod bipolar cells, identified by their characteristic morphology and PKC immunoreactivity (green), demonstrate an increase in pSTX3 immunoreactivity (red) under conditions of elevated intraterminal Ca^2+^ (“high Ca^2+^,” top panel) when compared to those incubated on a nominally 0 Ca^2+^ external solution (“low Ca^2+^,” bottom panel). Scale bar = 5 μm. **(B)** The mean pixel intensity of STX3 immunolabeling was not altered by changes in intraterminal Ca^2+^ (*p* = 0.7238). By contrast, elevation of intraterminal Ca^2+^ led to an increase in pSTX3 immunolabeling (*p* = 0.0077). **(C)** Quantification of the pSTX3/STX3 ratio in rod bipolar cell terminals indicates that elevated intraterminal Ca^2+^ (“High”) significantly increased this ratio above that of cells bathed in nominally O Ca^2+^ (“Low”). *p* < 0.0001. For **(B,C)**, *n* = 7,7 (mice). **(D)** Elevated intraterminal Ca^2+^ (“High”) also increased CaMKII activation, as evidenced by an increase in pCaMKII immunolabeling in synaptic terminals of rod bipolar cells relative to terminals bathed in nominally 0 Ca^2+^ external solution (“Low”). (*p* = 0.0283; *n* = 6,6 mice). *Indicates *p* < 0.05, **indicates *p* < 0.01, and ***indicates *p* < 0.001.

To probe the signaling pathway downstream of Ca^2+^ entry, we turned to the acutely isolated rod bipolar cell preparation (Zhou et al., 2006; Wan et al., 2008, 2010). This preparation allows us to manipulate intraterminal Ca^2+^ and examine Ca^2+^ signaling pathways in the absence of local circuit interactions (Wan et al., 2010, 2012). To elevate the level of free Ca^2+^ in the rod bipolar cell synaptic terminal, we used a low Na^+^ external solution to reverse the Na^+^/Ca^2+^ exchanger. This manipulation produces a modest, sustained elevation of the spatially-averaged intraterminal Ca^2+^ concentration (Wan et al., 2012). As shown in the representative PKC-positive terminal cluster of synaptic boutons of an individual RBC shown in Figure 5A (upper panel, “high Ca”), elevation of intraterminal Ca^2+^ via the reverse mode of the Na^+^/Ca^2+^ exchanger method produced a robust pSTX3 labeling that contrasted strongly with the minimal labeling observed in nominally Ca^2+^-free external solution (Figure 5A, lower panel, “low Ca”). Compiled data from seven experiments, with each experiment representing the average result obtained from numerous RBC terminals, showed that there was a significant increase in the pSTX3 immunolabeling in response to elevated intraterminal Ca^2+^ with no change in total STX3 levels (Figure 5B), suggestive of a Ca^2+^-dependent rise in phosphorylation of STX3 at T14. The mean pSTX3 to STX3 ratio in RBC synaptic terminals under conditions of elevated intraterminal Ca^2+^ was more than two-fold higher than those of cells bathed in a nominally Ca^2+^-free solution (Figure 5C; 0.944 ± 0.110 vs. 0.260 ± 0.052, *p* < 0.0001, *n* = 7,7), confirming this interpretation.

Given that STX3 is phosphorylated at T14 by CaMKII *in vitro* (Risinger and Bennett, 1999; Liu et al., 2014), we additionally asked whether CaMKII is activated via our stimulation paradigm. To this end, we performed parallel experiments in which immunolabeling for CaMKII at phosphorylated at T286/287 (pCaMKII) was used a read-out of constitutively-activated CaMKII (Colbran et al., 1989; Hudmon and Schulman, 2002). As shown in Figure 5D, elevation of intraterminal Ca^2+^ (“High”) produced an increase in pCaMKII labeling in RBC synaptic terminals relative to RBC terminals bathed in nominally Ca^2+^-free solution (“Low”) (High: 78.73 ± 7; Low: 54.99 ± 5.68; *t* = 2.562, *p* = 0.0283, *n* = 6,6).

### CaMKII Phosphorylates STX3 at T14 in the Rod Bipolar Cell

**FIGURE 6:**
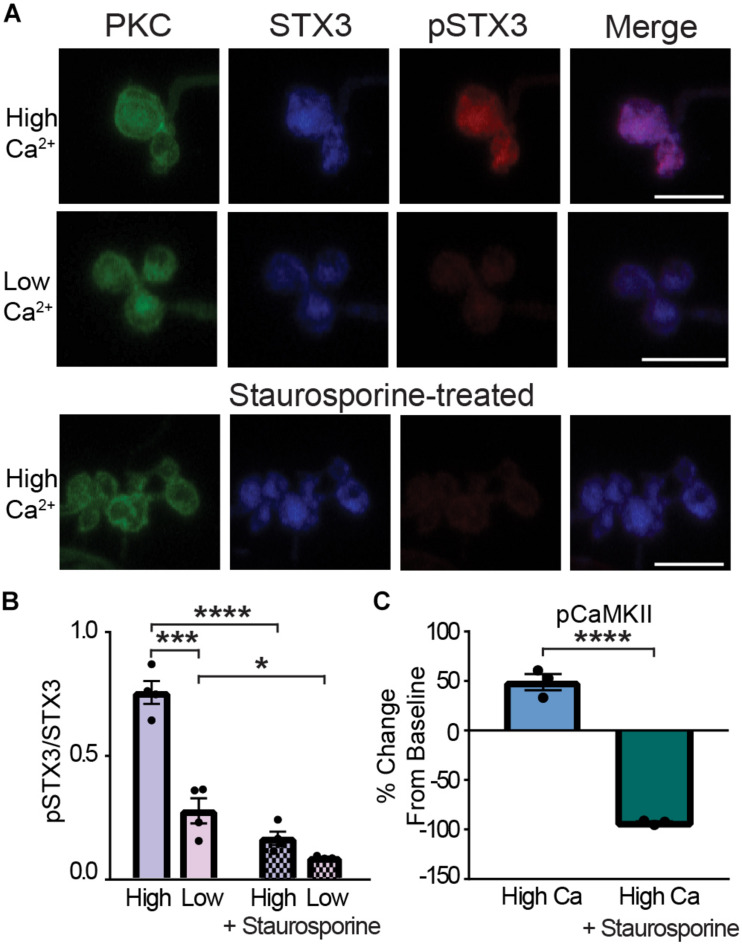
Kinase inhibition prevents the Ca^2+^-dependent phosphorylation of STX3B at T14 in rod bipolar cell synaptic terminals. **(A)** Confocal images of synaptic terminals of isolated rod bipolar cells, identified by their characteristic morphology and PKC immunoreactivity (green), demonstrate an increase in pSTX3 immunoreactivity (red) under conditions of elevated intraterminal Ca^2+^ (“High Ca^2+^”) when compared to those incubated in a nominally 0 Ca^2+^ external solution (“Low Ca^2+^”). Treatment with staurosporine (100 nM; lower panel) blocked the expected Ca^2+^-evoked increase in pSTX3 immunoreactivity. Scale bar = 5 μm. **(B)** Quantification of the pSTX3/STX3 ratio in rod bipolar cell terminals shows that elevated intraterminal Ca^2+^ (“High”) significantly increased the pSTX3/STX3 ratio above that of cells bathed in external solution containing no added Ca^2+^ (“Low”; *p* < 0.0001, *n* = 4,4 mice). Staurosporine not only blocked this Ca^2+^-evoked rise in the pSTX3/STX3 ratio (*p* < 0.0001, *n* = 4,4 mice), it lowered the ratio of the low Ca^2+^ control group (*p* = 0.0366, *n* = 4,3 mice). **(C)** In parallel experiments, treatment with staurosporine suppressed the Ca^2+^-stimulated increase in pCaMKII immunolabeling (*p* < 0.0001, *n* = 3,3 mice). *Indicates *p* < 0.05, ***indicates *p* < 0.001, and ****indicates *p* < 0.0001.

We next took advantage of our ability to manipulate Ca^2+^ and pSTX3 levels in the synaptic terminals of acutely isolated RBCs to test for a role for CaMKII. To validate this approach, we first ascertained whether inhibition of phosphorylation, in general, would prevent the Ca^2+^-mediated increase in pSTX3. To this end, acutely isolated RBCs were treated with the non-specific kinase inhibitor staurosporine (Figure 6). As shown in the images of representative RBC terminals (Figure 6A), treatment with staurosporine (100 nM) dramatically reduced both the Ca^2+^-triggered elevation of pSTX3 and the expected Ca^2+^- dependent rise in the pSTX3 to STX3 ratio. On average, the Ca^2+^-triggered increase in the pSTX3 to STX3 ratio was reduced by more than 75% with staurosporine treatment (Figure 6B; 0.167 ± 0.027 vs. 0.756 ± 0.046, *p* < 0.0001, *n* = 4,4). Furthermore, staurosporine lowered the residual pSTX3 to STX3 ratio of RBC synaptic terminals bathed in Ca^2+^-free solution (Figure 6B; 0.090 ± 0.004 vs. 0.278 ± 0.051, *p* = 0.0366, *n* = 4,3). Staurosporine treatment was also found to suppress the Ca^2+^-dependent increase in pCaMKII immunolabeling (Figure 6C).

To probe the ability of CaMKII to phosphorylate native STX3B at T14 in RBCs, we first turned to KN-93, a well-characterized, membrane-permeant CaMKII inhibitor that prevents the activation of CaMKII (Hudmon and Schulman, 2002). Treatment with KN-93 (1 μM, Supplementary Figure 2A) prevented the Ca^2+^-triggered elevation of the pSTX3 to STX3 ratio (high Ca: 0.944 ± 0.11; high Ca +KN-93: 0.375 ± 0.72; *p* = 0.0003, *n* = 7,7), holding the ratio at a level indistinguishable from that of unstimulated RBC terminals (0.260 ± 0.52, *p* = 0.996, *n* = 7,7). It also suppressed the Ca^2+^-induced increase in pCaMKII levels (Supplementary Figure 2B). However, treatment with 1 μM of KN-92 (Supplementary Figure 2A), an inactive analog of KN-93 that shares some but not all off-target effects with KN-93 (Pellicena and Schulman, 2014), also reduced the Ca^2+^-dependent rise in the pSTX3B to STX3B ratio and blocked the increase in CaMKII autophosphorylation (Supplementary Figure 2). These results raised the possibility that one or both compounds acted on targets other than or in addition to CaMKII, rendering it impossible to draw a conclusion about a possible *in vivo* role for CaMKII in STX3 phosphorylation using KN93.

**FIGURE 7:**
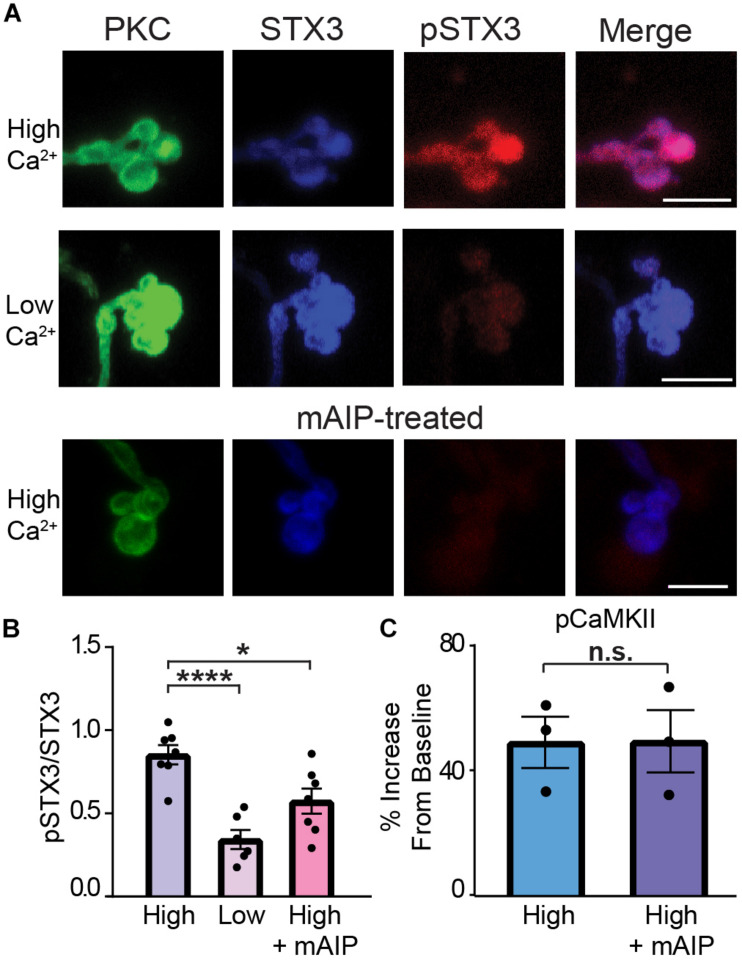
CaMKII phosphorylates STX3B at T14 in rod bipolar cell synaptic terminals. **(A)** Confocal images of synaptic terminals of isolated rod bipolar cells, identified by their characteristic morphology and PKC immunoreactivity (green), demonstrate an increase in pSTX3 immunoreactivity (red) under conditions of elevated intraterminal Ca^2+^ (“High Ca^2+^”) compared to those incubated in a nominally 0 Ca^2+^ external solution (“Low Ca^2+^”). Treatment with myristoylated AIP (“mAIP,” 100 nM, and lower panel) blocked the expected Ca^2+^-evoked increase in pSTX3 immunoreactivity. Scale bar = 5 μm. **(B,C)** Quantification of the pSTX3/STX3 ratio in rod bipolar cell terminals. AIP inhibited the Ca^2+^-evoked rise in the pSTX3/ST3 ratio (*p* = 0.0171 *n* = 7,7) without altering the Ca^2+^-evoked rise in pCaMKII immunoreactivity (*p* = 0.9791, *n* = 3,3 mice).

Therefore, to probe the role of CaMKII in STX3 phosphorylation at T14, we turned to the potent and highly selective peptide inhibitor of CaMKII called autocamtide-2-related inhibitory peptide (AIP; Ishida et al., 1995). AIP, derived from the autoinhibitory domain of CaMKII, inhibits all CaMKII activity regardless of activation mode (Ishida and Fujisawa, 1995; Ishida et al., 1995; Erickson et al., 2011; Pellicena and Schulman, 2014) and does not directly affect voltage-gated ion channels (Gao et al., 2006). Figure 7A shows representative examples of acutely dissociated PKC-positive RBC terminals under low and high Ca^2+^ conditions and RBCs treated with mAIP, a membrane-permeant analog of AIP, under high Ca^2+^ conditions. Incubation in external solution containing mAIP (100 nM) blocked the Ca^2+^-evoked increase in pSTX3. Pooled data from multiple experiments show that the Ca^2+^-triggered elevation of the pSTX3 to STX3 ratio was reduced in the presence of mAIP (Figure 7B; High: 0.852 ± 0.058; High + AIP: 0.573 ± 0.076, *p* = 0.0171, *n* = 7,7) to a ratio that was not dramatically different from that observed when external Ca^2+^ was nominally zero (low: 0.343 ± 0.058, *p* = 0.0616, *n* = 7,6). Interestingly, mAIP did not suppress the Ca^2+^-evoked elevation of pCaMKII in these experiments (Figure 7C). This suggests that the inhibitory effect of mAIP on STX3 phosphorylation observed here is most likely attributable to the ability of AIP to block constitutively-activated CaMKII (Ishida and Fujisawa, 1995; Erickson et al., 2011; Murakoshi et al., 2017).

### CaMKII Regulates Exocytosis at the Rod Bipolar Cell Synaptic Terminal

The above data are consistent with a model in which STX3 is phosphorylated at T14 in a Ca^2+^-dependent manner by CaMKII. To address the effect of this activity-dependent modification on synaptic function, we next examined the effect of CaMKII inhibition on RBC exocytosis. Membrane capacitance measurements were used to quantify exocytosis from an individual RBC in the retinal slice configuration held under voltage-clamp control. Exocytosis was triggered via a brief train of depolarizing voltage-pulses that depletes the ribbon-associated releasable vesicle pool (Wan et al., 2008, 2010; Wan and Heidelberger, 2011). To avoid modulating effects of AIP on targets outside of the rod bipolar cell under investigation, AIP [25 μM; (Sakaba and Neher, 2001; Tatone et al., 2002; Gao et al., 2006; Mockett et al., 2011)] was delivered directly to an individual rod bipolar cell via the whole-cell patch pipette. At this concentration, AIP is expected to selectively block both the autophosphorylation of CaMKII and activated CaMKII (Ishida et al., 1995). To allow time for both K^+^ channel blockers and AIP to reach the synaptic terminal from the somatically-positioned recording electrode, the stimulus was given approximately 15 min after achieving the whole-cell recording configuration. In addition, effectiveness of the delivery of blockers to the synaptic terminal was confirmed prior to stimulation by visual inspection of the current–voltage relationship and isolation of the presynaptic L-type Ca^2+^ current (Heidelberger and Matthews, 1992; Heidelberger et al., 2005; Wan and Heidelberger, 2011). Evaluation of the Ca^2+^ channel current–voltage relationship did not reveal a significant effect of AIP on the voltage-gated Ca^2+^ current either with respect to the mean activation voltage (Control: −42 ± 2 mV; AIP: −44 ± 1 mV; *p* = 0.4887, *t* = 0.7219; *n* = 6,5) or the mean Ca^2+^ current amplitude measured at −20 mV [Control: −23 ± 6 pA (median: −18 pA); AIP: −14 ± 2 pA (median: −16 pA); *p* = 0.2279, *t* = 1.343; and *n* = 6,5].

**FIGURE 8:**
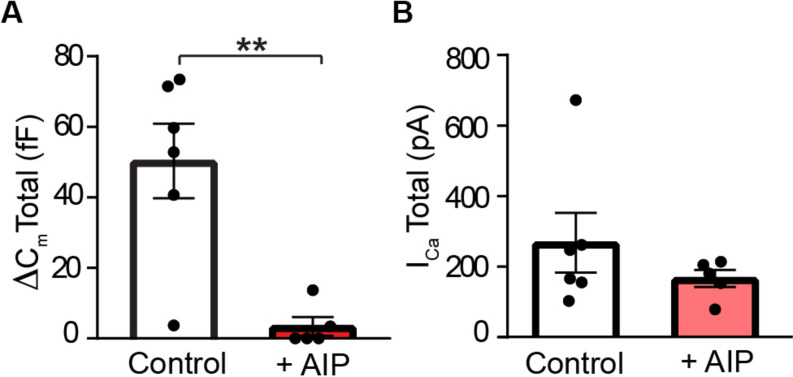
CaMKII inhibition suppresses exocytosis at rod bipolar cell terminals. **(A)** Total exocytosis, measured after ten depolarizing stimuli (−65 to −20 mV, 100 ms duration, 4 Hz; Wan et al., 2010), was significantly smaller when AIP (25 μM) was included in the internal recording solution than under control conditions (*p* = 0.0059). **(B)** The mean total I_Ca_, summed over the pulse train, was not significantly different in the absence or presence of AIP (*p* = 0.2948). For **A, B**, control: *n* = 6 cells/4 mice; AIP: *n* = 5 cells/3 mice.

As shown in Figure 8, control RBCs exhibited a mean depolarization-evoked capacitance increase at the end of the pulse train of ≈50 fF, a value slightly larger than the amplitude of the total releasable vesicle pool reported for dissociated mouse rod bipolar cells (Zhou et al., 2006; Wan et al., 2008). In the presence of AIP, however, the depolarization-evoked rise in membrane capacitance was reduced by approximately an order of magnitude (Figure 8A; Control: 50 ± 11 fF; AIP: 3.4 ± 2.7 fF; *p* = 0.0059, *t* = 4.309, and *n* = 6,5). These results indicate a major role for CaMKII in the regulation of exocytosis in RBC synaptic terminals. Because changes in exocytosis could also result from changes in Ca^2+^ entry, we additionally evaluated the effects of AIP on the train-evoked Ca^2+^ current. To correlate the total train evoked exocytosis with total train-evoked Ca^2+^ entry, we calculated the mean total Ca^2+^ current for the pulse train by taking the sum of each Ca^2+^ current evoked by an individual depolarizing voltage step within a stimulus train. As shown in Figure 8B, comparison of the total Ca^2+^ current for the pulse train failed to reveal a significant difference between control and AIP-treated rod bipolar cells [Control: −268 ± 85 pA (median value: −206 pA); AIP: −166 ± 24 pA (median value: −180 pA); *p* = 0.2948, *t* = 1.151; and *n* = 6,5]. This stimulation protocol and the levels of total Ca^2+^ current produced are sufficient to trigger fusion of the rapidly-releasable and releasable vesicle pools (Wan et al., 2008, 2012; Heidelberger, unpublished observations). While we cannot rule out the possibility that there could be differences in the intraterminal Ca^2+^ levels that might influence synaptic vesicle dynamics, the data suggest that AIP acts at a site other than Ca^2+^ entry to reduce exocytosis. A leading possibility, consistent with our previously published model (Liu et al., 2014), is that AIP inhibits the activity-dependent phosphorylation of STX3 at T14 by CaMKII, thereby modulating the assembly of SNARE complexes required for membrane fusion and the release of neurotransmitter.

## Discussion

Exocytosis at retinal ribbon-style synapses relies on a core fusion machinery that is formed between STX3 and SNAP-25, located on the plasma membrane, and VAMP/synaptobrevin, located on the synaptic vesicle (Morgans et al., 1996; Weber et al., 1998; Curtis et al., 2008, 2010; Rizo and Xu, 2015; Yoon and Munson, 2018). In this study, we utilized two well-characterized antibodies to quantify changes in STX3 phosphorylation at T14 in the synaptic terminals of retinal photoreceptors and bipolar cells. Results demonstrate for the first time that STX3 is phosphorylated *in vivo* at T14 in a light-regulated, Ca^2+^-dependent manner. Our results establish that the levels of T14 phosphorylation are higher under conditions associated with active neurotransmitter release than they are at rest. For example, in the synaptic terminals of the rod photoreceptor, the pSTX3 levels were highest in the dark-adapted state, when rods are depolarized and actively releasing neurotransmitter, while in the synaptic terminal of the rod bipolar cell, the pSTX3 levels were the highest following light exposure, consistent with the depolarized membrane potential of rod bipolar cells in the light. Experiments performed in the retinal eyecup preparation, which allowed for manipulation of the external milieu, provided clear evidence that the light-regulated increase in STX3 phosphorylation at T14 was dependent upon external Ca^2+^, consistent with activity-dependent Ca^2+^ entry, while experiments performed in the isolated rod bipolar cell demonstrated that elevation of intracellular Ca^2+^ alone was sufficient to promote STX3 phosphorylation at T14. Taken together, out results provide clear evidence that STX3 is phosphorylated at T14 *in vivo* in ribbon-style synapses of the rod pathway in response to the light-regulated, activity-dependent entry of Ca^2+^ and elevation of presynaptic Ca^2+^ levels.

In cone photoreceptor terminals the phosphorylation of STX3 at T14 also required external Ca^2+^, consistent with an activity-dependent mechanism (Figure 3). However, we did not detect a clear effect of illumination on STX3 phosphorylation *in vivo* or *ex viv*o. This may reflect the ability of cones to adapt and remain synaptically-functional under photopic conditions (Choi et al., 2005; Dowling, 2012; Ingram et al., 2019). That their synapses undergo exocytosis implies that the local Ca^2+^ concentration of at least some cone ribbon-style active zones is elevated. Thus, light-regulated alterations in the Ca^2+^-dependent phosphorylation of STX3 at T14 in cone terminals might be expected to be more subtle, on average, than those observed in rods and as such, not as readily-resolvable by quantitative immunofluorescence. This would be particularly true when the sample size is small as in the present study performed in mouse retina, where cones make up less than 3% of the total photoreceptor population (Jeon et al., 1998). Never-the-less, the clear Ca^2+^-dependence of STX3 T14 phosphorylation in cone terminals suggests that they also possess an activity-dependent regulatory pathway.

Our results provide the first evidence that CaMKII phosphorylates STX3 at T14 in a living neuron. CaMKII is also the only kinase predicted by sequence analysis to phosphorylate STX3 at T14 (Yaffe et al., 2001), and it is the only kinase tested that has been found to act on STX3 in controlled, biochemical experiments (Risinger and Bennett, 1999). Consistent with a role for CaMKII at retinal ribbon-style synapses, CaMKII has been localized to the synaptic terminals (Bronstein et al., 1988; Ullrich and Sudhof, 1994; Uthaiah and Hudspeth, 2010), where it has been affinity-purified with the synaptic ribbons as part of a ribbon-associated complex (Kantardzhieva et al., 2012). In addition, CaMKII has been shown to interact in a Ca^2+^-dependent manner with STX1A of conventional synapses in a region that is highly conserved with STX3B, raising the possibility that CaMKII may also directly associate with STX3B, the specific STX3 isoform expressed by retinal ribbon-style synapses (Ohyama et al., 2002; Curtis et al., 2008). For these reasons, CaMKII appears to be ideally situated to provide a link between light-regulated synaptic activity, Ca^2+^ entry and the modulation of STX3 phosphorylation at T14.

The specific isoform of CaMKII responsible for the phosphorylation of STX3 at T14 *in vivo* is not yet known. The pCaMKII T286/287 antibody that produced robust synaptic labeling in stimulated rod bipolar cells terminals recognizes all four isoforms of phosphorylated CaMKII. In agreement with a previous report (Tetenborg et al., 2017), antibodies directed against CaMKIIα failed to immunolabel rod bipolar cell terminals (not shown), suggesting that an isoform other than CaMKIIα is likely to be involved. Interestingly, CaMKII2δ has been localized to the synaptic terminals of rod bipolar cells (Tetenborg et al., 2017), and analysis of isolated mouse retina ribbons has revealed a higher level of CaMKII2δ relative to other CaMKII isoforms in the ribbon-associated complex (Kantardzhieva et al., 2012). Taken together, these findings suggest that CaMKIIδ may phosphorylate STX3 at T14 at the ribbon-style synapses of rod photoreceptors and rod bipolar cells. However, additional studies, beyond the scope of the present study, would be required to test this possibility and to evaluate the extent to which other kinases, not yet examined, may also act on this site *in vivo*.

This study also provides the first functional evidence consistent with a role for the T14 phosphorylation site of STX3 in the regulation of exocytosis. Our results in rod bipolar cells demonstrate that the highly selective CaMKII inhibitor AIP produced a dramatic reduction in stimulus-evoked exocytosis in the absence of a significant alteration in voltage-dependent Ca^2+^ entry. Given that AIP was delivered selectively to a single rod bipolar cell under investigation, local circuit interactions were blocked, and exocytosis was monitored via changes in membrane capacitance in that same rod bipolar cell, the AIP-induced decrease in exocytosis must arise from a change in functionality within that same rod bipolar cell. Furthermore, because the stimulus used triggers the fusion of the ribbon-associated vesicle pool (Zhou et al., 2006; Wan et al., 2008; Wan and Heidelberger, 2011), the observed inhibition of exocytosis is consistent with a local action of CaMKII at the ribbon-style active zone rather than more broadly throughout the cell. With respect to the other members of the core fusion machinery only synaptobrevin/VAMP has been consistently shown to be *in vitro* phosphorylated by CaMKII (Nielander et al., 1995; Hirling and Scheller, 1996; Risinger and Bennett, 1999). But in contrast to STX3, modification of the synaptobrevin/VAMP CaMKII phosphorylation sites does not appear to effect SNARE complex assembly or exocytosis (Hirling and Scheller, 1996; Regazzi et al., 1996). In addition, a phosphoproteomics approach applied to isolated brain synaptosomes found no evidence for activity-dependent phosphorylation of either synaptobrevin/VAMP or SNAP-25 or of the syntaxin used by conventional synapses, STX1 (Kohansal-Nodehi et al., 2016). Interestingly, STX1 is not a CaMKII substrate but may be constitutively phosphorylated (Hirling and Scheller, 1996; Risinger and Bennett, 1999; Foletti et al., 2000). Thus, not only may STX3 regulate release in response to synaptic demand via a Ca^2+^- and CaMKII-dependent pathway, it appears to be unique in this regard.

How might STX3 phosphorylation at T14 modulate release? We have shown previously that a phosphomimetic T14 mutation of STX3 interacts more readily with its SNARE binding partner SNAP-25 than wild-type STX3 (Liu et al., 2014), suggesting a role for T14 phosphorylation in STX3 activation and the regulation of SNARE complex assembly. Furthermore, residue 14 of STX resides within a region that has a critical interaction with Munc18, which in STX1 stabilizes the STX open conformation and facilitates SNARE complex formation and exocytosis (Khvotchev et al., 2007; Rathore et al., 2010; Shen et al., 2010; Christie et al., 2012; Zhou et al., 2012). Subtle charge changes in this region, such as by phosphorylation, are predicted to alter this critical interaction and influence exocytosis (Rickman and Duncan, 2009; Shen et al., 2010; Christie et al., 2012; Colbert et al., 2013; Shi et al., 2020). Our current finding that inhibition of CaMKII, which suppresses the Ca^2+^-dependent phosphorylation of STX3 at T14, modulates stimulus-evoked exocytosis aligns well with these studies. However, additional studies will be needed to directly probe of the role(s) of STX3 T14 phosphorylation/dephosphorylation in the regulation of exocytosis.

Important for all neurons, Ca^2+^-dependent modulation of the secretory pathway is particularly relevant to the visual, auditory and vestibular systems, where information about the outside world, covering many log units of dynamic range, is encoded in a largely analog fashion by ribbon-style synapses. To maintain this ability, these synapses must efficiently replace vesicles lost to stimulus-evoked fusion with new, fusion-competent (primed) vesicles. Studies in retinal bipolar cells going back more than a decade have shown that the delivery of vesicles to the fusion-competent state is accelerated by the elevation of presynaptic Ca^2+^ (Mennerick and Matthews, 1996; Gomis et al., 1999; Singer and Diamond, 2006; Wan et al., 2010), however, the molecular mechanism(s) has remained elusive. At conventional synapses, two Ca^2+^-dependent processes regulate the availability of vesicles for release. The first involves a vesicle-associated phosphoprotein called synapsin that tethers synaptic vesicles in the cytosol to the actin cytoskeleton (De Camilli and Greengard, 1986; Cesca et al., 2010). In response to synaptic activity, CaMKII phosphorylates synapsin, freeing the vesicles from the cytoskeleton and allowing them to replenish the supply of active zone vesicles (De Camilli and Greengard, 1986; Benfenati et al., 1992; Cesca et al., 2010). The second mechanism involves Munc13. Essential for conventional synapse function, Munc13 catalyzes a required conformational change in STX1 that is permissive for SNARE complex assembly (Ma et al., 2011). Munc13 is not a CaMKII substrate, but has binding sites for Ca^2+^ and calmodulin and has been implicated in the activity-dependent preparation of vesicles for fusion and in Ca^2+^-dependent short-term plasticity at conventional synapses (Varoqueaux et al., 2002; Lipstein et al., 2013). In this regard, retinal ribbon-style synapses differ in two important ways. First, retinal ribbon-style synapses do not express synapsins (Mandell et al., 1990; Ullrich and Sudhof, 1994), an important CaMKII target of conventional synapses, and consistent with a lack of synapsins, cytosolic vesicles in retinal ribbon-style synapses are freely mobile (Holt et al., 2004). Secondly, Munc13 proteins are reportedly not required for exocytosis (Cooper et al., 2012). Together, this raises the question of what regulates STX3 activation, SNARE complex assembly and the Ca^2+^-accelerated progression to the fusion competent state in a retinal ribbon-style synapse. Intriguingly, both Ca^2+^ and calmodulin have been suggested to speed the delivery and attachment of synaptic vesicles to the synaptic ribbons (Van Hook et al., 2014). However, this action would not necessarily confer fusion competence. An additional action on SNARE complex assembly would be required. Our results suggest that the Ca^2+^-dependent phosphorylation of STX3B at T14 by CaMKII may subserve this function.

STX3 has two major splice forms, STX3A and STX3B. STX3A and STX3B are virtually identical from the N-terminus through approximately the middle of the SNARE binding domain, differing only in the second half of the SNARE domain and the transmembrane domain due to the use of different exons in the 3’ regions of the two mRNAs (Curtis et al., 2008). Thus, they share the identical T14 phosphorylation site. Given that STX3B expression is restricted to the retina, and in particular photoreceptors and bipolar cells, and there is no appreciable STX3A in mouse retina (Curtis et al., 2008), the current results likely reflect the light-regulated, Ca^2+^-dependent phosphorylation of STX3B. STX3A, on the other hand, is widely expressed throughout the body, where it plays roles in exocytosis and apical trafficking, particularly within the immune, renal and digestive systems (Lehtonen et al., 1999; Torkko et al., 2008; Zhu et al., 2013; Brochetta et al., 2014; Wiegerinck et al., 2014; Julia et al., 2019). Given the conservation of the T14 site between STX3A and STX3B, these essential processes may also be modulated via the Ca^2+^-dependent phosphorylation of STX3 at T14. Finally, we note that neither STX3 isoform has been reported at the ribbon-style synapses of cochlear hair cells (Uthaiah and Hudspeth, 2010). This suggests an additional level of synaptic specialization that tailors the secretory machinery utilized by ribbon-style synapses to the meet the challenges of their particular sensory systems.

## Data Availability Statement

The raw data supporting the conclusions of this article will be made available by the authors, without undue reservation.

## Ethics Statement

The animal study was reviewed and approved by Animal Welfare Committee of The University of Texas Health Science Center at Houston.

## Author Contributions

RH, HL, and RJ conceived the presented idea and designed the experiments. JC, HL, YW, XL, MK, and AH conducted experiments. All authors analyzed data and contributed to interpretation. JC, RH, and AH prepared the manuscript with input from all authors.

## Conflict of Interest

The authors declare that the research was conducted in the absence of any commercial or financial relationships that could be construed as a potential conflict of interest.
