# Supplementary material for: Phosphorylation of the Retinal Ribbon Synapse Specific t-SNARE Protein Syntaxin3B Is Regulated by Light via a Ca2 +-Dependent Pathway
Source: Front Cell Neurosci. 2020 Oct 20;14:587072. doi: 10.3389/fncel.2020.587072 (PMC7606922; doi:10.3389/fncel.2020.587072)
Supplement: Supplementary file 1 [file Data_Sheet_1.PDF]

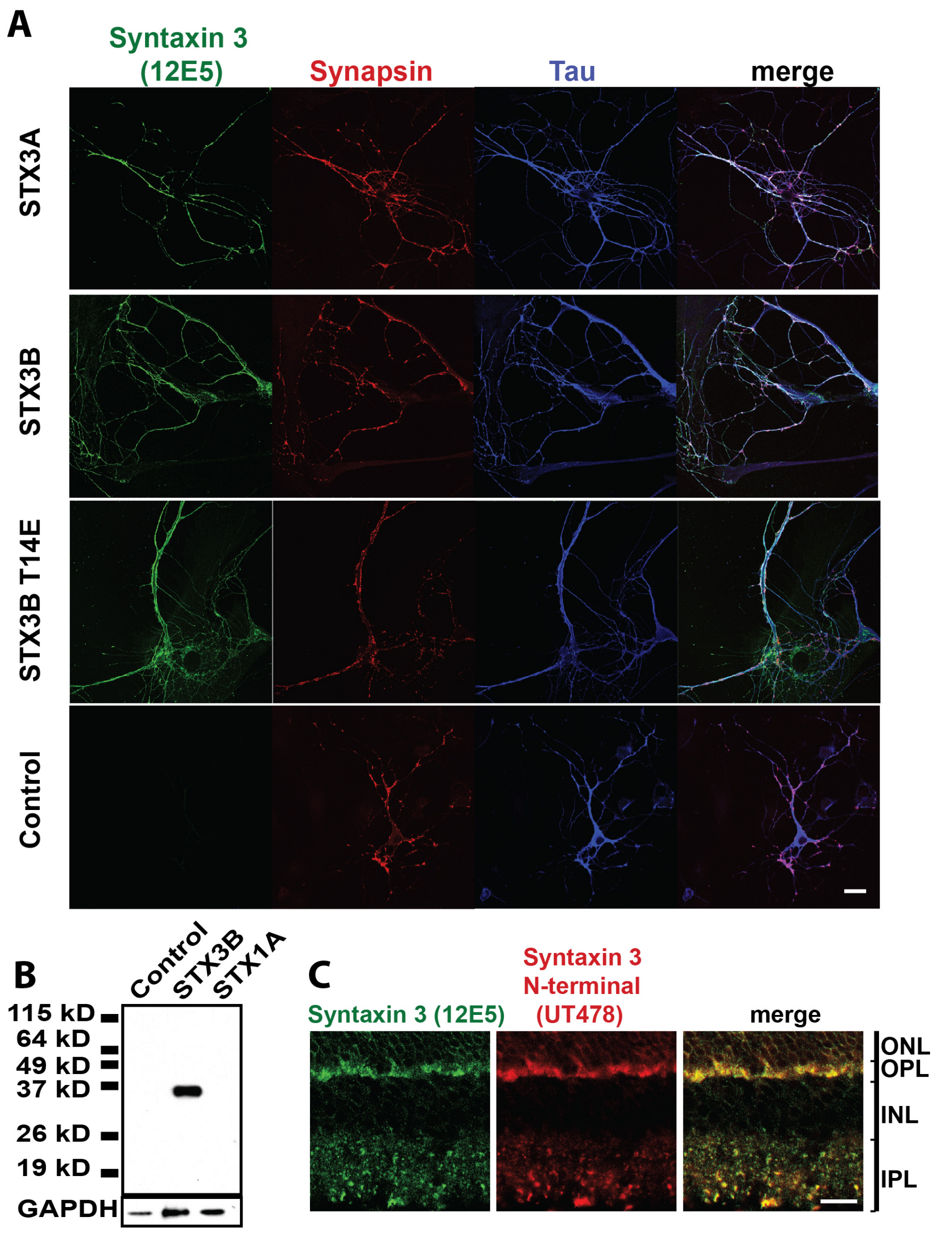

Supplementary Figure 1. Validation of the specificity of the 12E5 mouse monoclonal syntaxin 3 antibody. (A) The 12E5 syntaxin mouse monoclonal antibody recognizes syntaxin 3A (STX3A), syntaxin 3B (STX3B) and syntaxin 3B that contains the T14E phosphomimetic mutation (STX3B T14E). Cultured primary mouse hippocampal cultures, which do not express detectable levels of syntaxin 3, were transduced with mouse syntaxin 3A, mouse syntaxin3B, or mouse syntaxin 3B containing the phosphomimetic T14E mutation using a lentiviral system (Govorunova et al., 2017). Neurons were triple-labeled for syntaxin 3 using the 12E5 syntaxin 3 antibody, synapsin (to mark the conventional synapses) and tau (an axonal marker). The syntaxin 3 12E5 mouse monoclonal antibody recognized both syntaxin 3A and 3B. In addition, labeling of syntaxin 3B was not affected by the T14E phosphomimetic mutation. As expected, un-transduced cells exhibited only extremely weak background labeling for syntaxin 3 (control).

Scale bars: 20μm. (B) The 12E5 syntaxin 3 antibody does not cross-react with syntaxin 1A. Mouse syntaxin 3B and syntaxin 1A were expressed in cultured primary mouse hippocampal neurons as described above, and cell extracts were analyzed by SDS-PAGE and western blotting using the 12E5 syntaxin 3 antibody. Samples were also analyzed using a GAPDH antibody as a loading control (bottom panel). The antibody reacts with a protein of the predicted size in the extracts from cells expressing syntaxin 3B but not in the un-transduced cells (control) or in cells overexpressing syntaxin 1A, demonstrating strong isoform specificity. (C) Binding of the 12E5 mouse monoclonal syntaxin 3 antibody does not interfere with the binding of an N-terminal syntaxin 3 antibody. Vertical sections of mouse retina were double-labeled with the 12E5 syntaxin 3 antibody and a previously characterized rabbit polyclonal syntaxin 3 antibody (UT478) raised against an N-terminal peptide epitope (amino acids 2-17) of syntaxin 3 (Liu et al., 2014). The labeling obtained with the two antibodies was strongly co-localized. Similar results were obtained at different concentration ratios of the two antibodies. These findings demonstrate that there is no interference in the binding of the two antibodies and that the epitope recognized by the 12E5 antibody is not located within the N-terminal region where T14 resides. Scale bars: 20μm

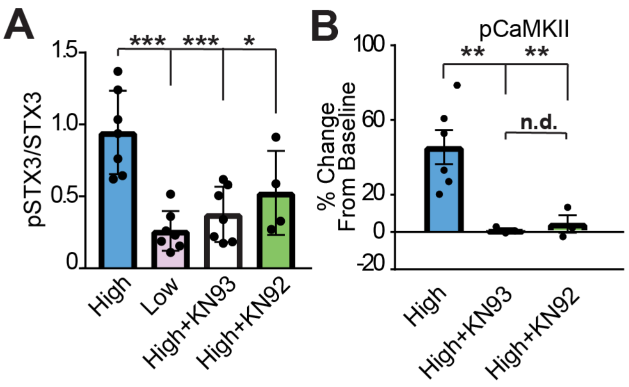

Supplementary Figure 2: KN-93 and KN92 attenuated the Ca^{2+}-evoked phosphorylation of STX3B at T14 in rod bipolar cell synaptic terminals. (A) Comparison of the pSTX3/STX3 ratio in the synaptic terminals of isolated rod bipolar cells under difference conditions. Elevated intraterminal Ca^{2+} ("High') significantly increased the pSTX3/STX3 ratio above that of cells bathed in external solution containing no added Ca^{2+} ("Low") ($p<0.0001$, n=7,7 mice). Both KN-93 and KN-92 (an analog of KN-93 that does not act on CaMKII and has different off-target effects) attenuated the Ca^{2+}-evoked rise in the pSTX3/ST3 ratio relative to elevated Ca^{2+} alone (KN93: p=0.0007 n=7,7; KN92: p=0.0373 n=7,4). (B) Both KN-93 and KN-92 reduced the Ca^{2+}-evoked phosphorylation of CaMKII at T286/287 as indicated by the reduction of the Ca^{2+}-evoked percent increase in pCaMKII immunoreactivity relative to high Ca^{2+} alone (KN93: p=0.0131, n=6,3; KN92: p=0.0199, n=6,3). Thus, one or both reagents may act upstream of CaMKII activation, rendering it difficult to draw a conclusion about the roles of CaMKII in STX3 phosphorylation from these data. For both A and B, each data point represents the mean value obtained from analysis of multiple synaptic terminals from an individual mouse.

Supplementary References

Govorunova EG, Sineshchekov OA, Rodarte EM, Janz R, Morelle O, Melkonian M, Wong GK, Spudich JL. The expanding family of natural anion channelrhodopsins reveals large variations in kinetics, conductance, and spectral sensitivity. *Science Reports*, 7:43358, 2017. PMCID: PMC5335703

Liu X, Heidelberger R, and Janz R. Phosphorylation of syntaxin 3B by CaMKII regulates the formation of a t-SNARE complex. *Molecular and Cellular Neuroscience,* 60:553-562, 2014. PMCID: PMC4066811
